# Stability Analysis of Anthocyanins Using Alcoholic Extracts from Black Carrot (*Daucus Carota* ssp. *Sativus* Var. *Atrorubens* Alef.)

**DOI:** 10.3390/molecules23112744

**Published:** 2018-10-24

**Authors:** Guillermo Espinosa-Acosta, Ana L. Ramos-Jacques, Gustavo A. Molina, Jose Maya-Cornejo, Rodrigo Esparza, Angel R. Hernandez-Martinez, Itari Sánchez-González, Miriam Estevez

**Affiliations:** 1 Posgrado en Ciencia e Ingeniería de Materiales, Centro de Física Aplicada y Tecnología Avanzada (CFATA), Universidad Nacional Autónoma de México (UNAM), Blvd. Juriquilla 3000, Querétaro, Mexico; senz_ad@hotmail.com (G.E.-A.); gustavomolina21@gmail.com (G.A.M.); 2 Independent Researcher, El Canto, Zibatá 76269, Querétaro, Mexico; al.ramos.jacques@gmail.com; 3 Centro de Física Aplicada y Tecnología Avanzada (CFATA), Universidad Nacional Autónoma de México (UNAM), Blvd. Juriquilla 3000, Querétaro, México; iqm_jamc@yahoo.com.mx (J.M.-C.); resparza@fata.unam.mx (R.E.); arhm@fata.unam.mx (A.R.H.-M.); 4 Licenciatura en Tecnología, Centro de Física Aplicada y Tecnología Avanzada (CFATA), Universidad Nacional Autónoma de México (UNAM), Blvd. Juriquilla 3000, Querétaro, México; itari_13@hotmail.com

**Keywords:** anthocyanin, natural extract, tetraethyl orthosilicate, black carrot, antioxidant activity

## Abstract

Anthocyanins are used for food coloring due their low toxicity and health benefits. They are extracted from different sources, but black carrot has higher anthocyanin content compared with common fruits and vegetables. Here, we study alcoholic anthocyanin extracts from black carrot to enhance their stability. The objective of our research is to determine if microencapsulation with tetraethyl orthosilicate (TEOS) is a feasible option for preventing black carrot anthocyanin degradation. Extraction solvents were solutions of (1) ethanol/acetic acid and (2) ethanol/citric acid. Samples were purified through a resin column and microencapsulated using TEOS. Fourier Transformed Infrared Spectroscopy (FTIR) spectra of samples were obtained, and degradation studies were performed under different conditions of UV radiation, pH and temperature. Antioxidant activity was evaluated with radical 2,2-diphenyl-1-picrylhydrazyl (DPPH) scavenging and electrochemical cupric reducing antioxidant capacity (CUPRAC). Color evaluation on food models were performed with CIE Lab at the beginning of experiments and after 25 days of storage. Results indicate that the more stable extracts against pH media changes are samples obtained with ethanol/acetic acid solution as extraction solvent. Extract purification through resin and TEOS microencapsulation had no significant effect on extract stability. In conclusion, although TEOS microencapsulation has proven to be effective for some dried materials from natural extracts in our previous research, we do not recommend its use for black carrot extracts considering our results in this particular case.

## 1. Introduction

The use of extracts from natural sources as food coloring is an ongoing trend because, in general, they are Generally Recognized as Safe (GRAS) substances and bring health benefits for consumers [1]. A variety of natural colorants are used in the food industry, but there is still a concern about their production costs and stability and their performance had been studied for the past decade [2,3,4,5,6]. Substances like carotenoid, chlorophyll, turmeric, anthocyanin, and betalain extracts from natural sources impart a variety of colors [7,8].

Our hypothesis relies on the possibility of using microencapsulation (with TEOS) to increase stability of anthocyanins. Previously, we studied betalain extracts from *Beta vulgaris* and *Myrtillocactus geometrizans* and obtained dried materials that were microencapsulated using TEOS, obtaining an improvement in the materials’ stability against UV light, pH and temperature [1]. 

Anthocyanins (polyhydroxy and polymethoxy derivatives of 2-phenylbenzopyrylium of flavylium salts) are a group of phenolic compounds that are responsible for the colors of flowers, fruits, and vegetables [9]. It has been reported that this group of compounds has antioxidant [10], antimutagenic, anticancer and antiobesity properties, and they reduce the risk of coronary heart disease [9,11,12]. The colors imparted by anthocyanins are bright and could be used in the food industry as a replacement for colorants like FD&C Red 40 [13]. The colors obtained are commonly red, orange, blue and purple, depending on the chemical structure of anthocyanins; but, structural transformations are induced by changes in the pH of the medium, that affect both color quality and intensity [14]. 

Common sources of anthocyanins are purple corn, red cabbage, purple sweet potato, apples, grapes, kiwi, red onions and several berries [6,15]. Some studies have used local plants like black carrot [6]. Black or purple carrot (*Daucus carota* ssp. *sativus* var. *atrorubens* Alef.) was originally from Turkey and the Middle and Far East [16], but recently, new varieties with high anthocyanin content had been cultivated in other parts of the world [12,13,16]. For those reasons, this source was selected for our study.

The stability of anthocyanins in plant extracts depends on the temperature and solids content; and by increasing these conditions, the degradation rates of anthocyanins increase too. To minimize this degradation, it is recommended to cool the concentrates as soon as produced. Other factors that affect color and stability of anthocyanins are concentration, light, presence of co-pigments, metallic ions, enzymes, sugars, proteins, and antiradical activity (which quantifies the ability of complex chemical structures to scavenge free radicals) [2,4,17,18,19,20,21].

Considering those factors, many efforts have been made to enhance anthocyanins’ stability. Acylation of anthocyanins is the most commonly used method, as it has been reported that acylated cyanidin derivatives are more stable during prolonged storage compared to the corresponding non-acylated ones [12,13,16] Stintzing et al. [10] also confirmed that there is an increase in color strength through acylation. On the other hand, microencapsulation has been used to increase stability of natural colorants (anthocyanin and betalain derivatives) [2,4,17,18,19]. Then, considering these conditions, some efforts have been made to preserve natural extracts and colorants using different techniques, such as removing compounds through resin columns and microencapsulation with TEOS.

Here, we study the stability of black carrot extracts while modifying the experimental conditions, including extraction solvents, and we study the feasibility of microencapsulation with TEOS and the repercussion of this procedure on the stability of the extracts. 

## 2. Results and Discussion

### 2.1. Anthocyanin Content 

**Figure 1:**
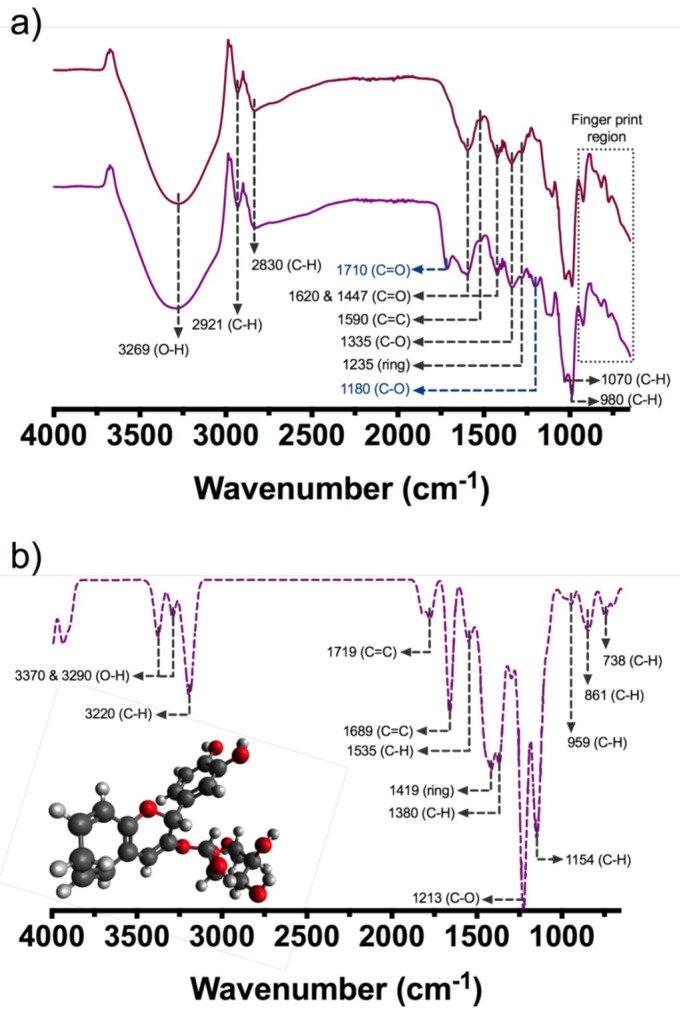
(**a**) Experimental FTIR spectra of black carrot extracts; samples extracted with ethanol/citric acid (BCS) and ethanol/acetic acid (BCA), (**b**) Simulated FTIR spectra using B3LYP calculation and cyanidin 3-O-glucoside molecule (used for simulation).

Major groups of substances were quantified with an UV-Vis spectroscopy analytic method. Figure 1 shows the Fourier Transformed Infrared (FTIR) spectra of samples extracted with ethanol/citric acid (BCS) and ethanol/acetic acid (BCA) and the IR spectra obtained by simulation. In Figure 1a, vibrations obtained for both samples showed a band at 980 cm^−1^ of an C-H in plane deformation, at 1070 cm^−1^, corresponding to an aromatic ring C-H deformation, bands at 1620 and 1447 cm^−1^, that correspond to vibration (C=O) of the benzopyran aromatic ring and 1590 cm^−1^ from the stretching vibration (C=C) of an aromatic ring, a band at 1235 cm^−1^, that corresponds to stretching of pyran rings, typical of flavonoid compounds, and a band at 1335 cm^-1^, that corresponds to C-O angular deformations of phenols, at 2830 and 2921 cm^−1^ due to symmetric and asymmetric C-H vibration respectively and 3269 cm^−1^ from O-H stretching vibration. In BCS samples there are two additional peaks, at 1710 cm^−1^ from a C=O stretching vibration and a 1180 cm^−1^ that correspond to C-O symmetric vibration, this is indicative that there are other acyl compounds in the extract that are account for a major proportion on the surface.

Becke 3-Lee-Yang-Parr (B3LYP) model simulation results are shown in Figure 1b, considering the cyanidin 3-O-glucoside molecule. The bands obtained are 3370 and 3290 cm^−1^ (O-H symmetric stretching vibration), 3220 cm^−1^ (C-H symmetric stretching), 1719 and 1689 cm^−1^ (C=C scissoring on pyran and phenolic group respectively), 1535 cm^−1^ (C-H scissoring), 1419 cm^−1^ (asymmetric ring vibration on plane), 1380 cm^−1^ (C-H deformation), 1213 cm^−1^ (C-O stretching), 1154 cm^−1^ (a scissoring plane vibration of phenol ring), 959 cm^−1^ (phenol ring C-H asymmetric stretching), 861 cm^−1^ (C-H phenol ring symmetric stretching) and 738 cm^−1^ (phenol ring C-H deformation).

**Figure 2:**
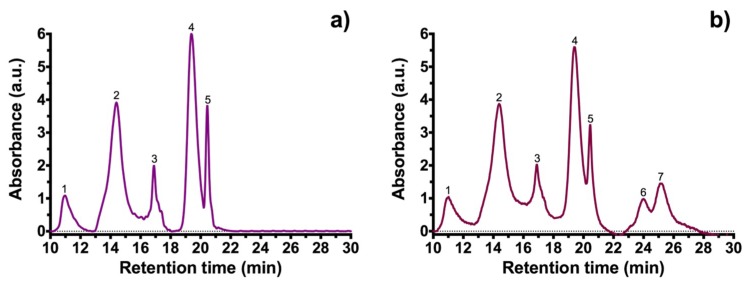
High performance liquid chromatography (HPLC) of black carrot extracts: (**a**) sample BCS (extracted with ethanol/citric acid). (**b**) sample BCA (extracted with ethanol/acetic acid). Peaks were identified and are shown in Table 1.

These results suggest that cyanidin 3-O-glucoside is the major anthocyanin component in black carrot, since they share similar vibrational frequencies and the same functional groups, like previously reported values for black carrot [20,21,22,23]. To confirm this notion, high performance liquid chromatography (HPLC) was performed for samples BCS and BCA. Figure 2a shows the HPLC chromatogram from sample BCS and Figure 2b shows the HPLC chromatogram for BCA. Peak identification was made using previous reports [22,23,24]. Table 1 shows the retention times of the characteristic peaks and compounds identified.

The chromatogram of sample BCS shows two additional peaks (anthocyanins reported as derived from feluric acid) compared with sample BCA. HPLC analysis confirmed the presence of cyanidin 3-O-glucoside molecule and its derivatives in the black carrot extracts obtained.

**Figure 3:**
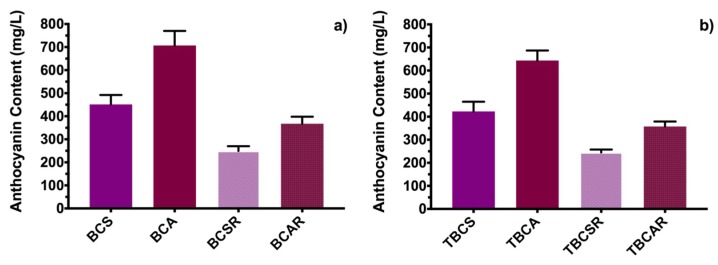
Anthocyanin content in black carrot extracts. (**a**) Samples extracted with ethanol/citric acid (BCS) and ethanol/acetic acid (BCA), and samples after resin column (BCSR and BCAR). (**b**) Microencapsulated samples using TEOS (TBCS, TBCA, TBCSR, TBCAR).

Figure 3 shows the total anthocyanin content obtained by a differential pH analytical method. Using ethanol with acetic acid as extraction solvent (sample BCA) leads to more anthocyanin content compared with using ethanol and citric acid, which is congruent with results published before on anthocyanin quantification of extracts from other plants [24,25]. Anthocyanin content is reduced significantly after passing through an Amberlite XAD7 resin column (Figure 3a); this could be explained as a natural degradation process under the experimental conditions. Further chromatographic studies should investigate if 3-O-glucoside anthocyanin derivatives could be trapped in the resin.

The four extracts (BCA, BCS, BCAR, and BCSR) were microencapsulated using TEOS and their anthocyanin content is shown in Figure 3b; there was an average loss of 7 ± 0.1% and 2.5 ± 0.005% in the anthocyanin content for samples without passing through the resin column (TBCS, TBCA) and samples after passing through the resin column (TBCSR, TBCAR), respectively. That suggests that TEOS incorporation has a negligible effect in preventing degradation. This could be explained by hydrolysis and condensation processes of the alkoxide that could be favored by functional groups of the acids used. Also, bonds between Si-O and 3-O-glucoside structure, as proposed by other authors [1,3], could lead to less total anthocyanin content in microencapsulated samples than in samples without microencapsulation. 

The anthocyanin contents for BCS, BCSR and BCAR and the same microencapsulated samples are in accordance with other microencapsulated powder samples reported for black carrot extracts [8]. BCA and TBCA extracts had the highest anthocyanin content compared with previously mentioned extracts, but it was not as high as the extraction reported using enzymes for other subspecies of black carrot [12,18], nevertheless the extraction method reported here, is inexpensive compared with others and it could be competitive for several industrial applications.

### 2.2. UV Radiation Study

**Figure 4:**
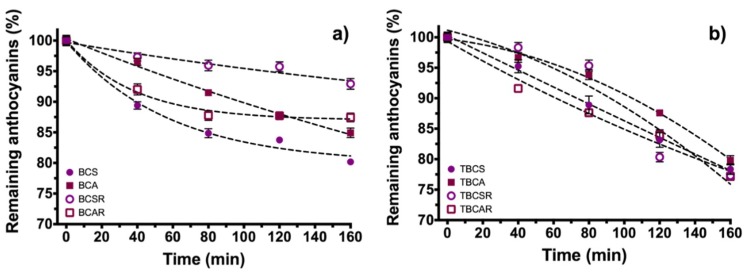
Anthocyanin content in black carrot extracts as function of time under UV radiation (315–400 nm); (**a**) Samples extracted with ethanol/citric acid (BCS) and ethanol/acetic acid (BCA), and samples after resin column (BCSR and BCAR). (**b**) Microencapsulated samples using TEOS (TBCS, TBCA, TBCSR, TBCAR).

In the UV radiation stability test (Figure 4a), there was a reduction of 19.81% and 17.99% of the total anthocyanin content for BCS and BCA, respectively. On the other hand, the extracts under resin purification BCSR and BCAR had a reduction of 7.06% and 12.55% from its total anthocyanin content. Considering statistical variations, it cannot be ensured that anthocyanin acylation results in protection against UV radiation.

Figure 4b shows samples microencapsulated with TEOS, and they follow a quasi-linear decay behavior instead an exponential decay (non-microencapsulated extracts). This can be seen in the reduction of total anthocyanin content which was 21.63%, 20.17%, 22.68%, and 22.82% for TBCS, TBCA, TBCSR and TBCAR, respectively, that are higher losses compared with samples without microencapsulation. 

### 2.3. Thermal Stability

**Figure 5:**
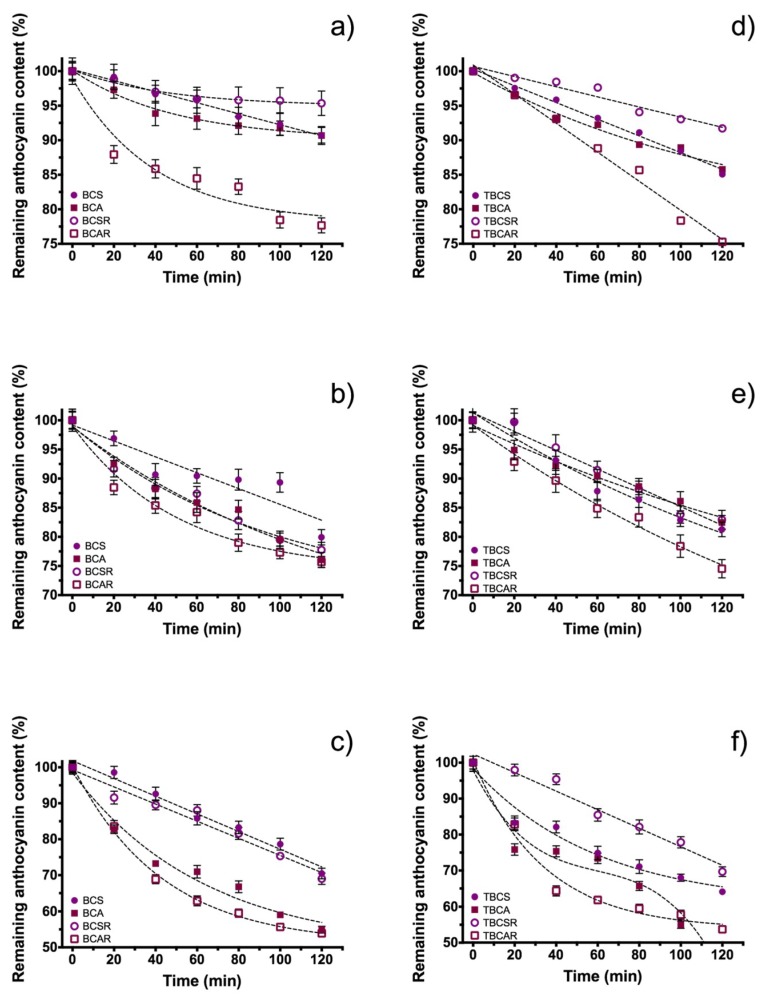
Anthocyanin content as function of time at different temperatures; (**a**,**d**) at 40 °C, (**b**,**e**) at 60 °C, and (**c**,**f**) at 80 °C. Samples extracted with ethanol/citric acid (BCS) and ethanol/acetic acid (BCA), samples after resin column (BCSR and BCAR), and microencapsulated samples using TEOS (TBCS, TBCA, TBCSR, TBCAR).

Sample BCA presents same trend compared with BCAR in thermal stability at different temperatures (Figure 5a–c). The same behavior was obtained for BCS and BCSR, therefore purifying extracts through resin column does not have a significant improvement in thermal stability. There is a difference in the behavior of the decay between extracts obtained with ethanol/acetic acid and ethanol/citric acid as extraction solvent; the first ones have a greater decay rate because their graphs (Figure 5) have a greater slope. This slope difference is more visible at high temperatures. On the other hand, considering anthocyanin content loss percentages of 9.28% and 9.31% at 40 °C, 20.08% and 23.96% at 60 °C, and 29.53% and 47.07% at 80 °C for BCS and BCA, respectively. The thermal stability behavior of anthocyanins as a function of time at 40 and 60 °C for TBCA and TBCAR samples is similar (Figure 5d,e). TBCS and TBCSR also had the same trend in thermal stability curves at 40 and 60 °C, from this behavior we assume that the resin column did not influence the thermal stability. Nevertheless, at 80 °C ethanolic extraction with acetic acid and encapsulated samples showed a rapid decay in anthocyanin content. Comparing the total anthocyanins loss of the encapsulated and non-encapsulated samples (9.28% and 14.93% for BCS and TBCS; 9.31% and 8.2% for BCA and TBCA, respectively) at 40 °C, it is possible to conclude that microencapsulation does not prevent thermal degradation in this case. In higher temperatures the same conclusion was obtained (for example at 80 °C: 29.53% and 35.91% for BCS and TBCS, 45.07% and 57.15% for BCA and TBCA, respectively).

These results showed that the anthocyanins were more stable using ethanol with citric acid (as extraction solvent) and purified with resin at 40 °C (BCSR) and the stability can be slightly enhanced when TEOS microencapsulation is used at high temperatures. In this experiment, BCAR (extraction of ethanol acidified with acetic acid after purification) had the highest value of degradation and it is more clearly when microencapsulation with TEOS is used. For samples where resin is used, the results are consistent, even using TEOS for microencapsulation.

### 2.4. pH Storage Stability

**Figure 6:**
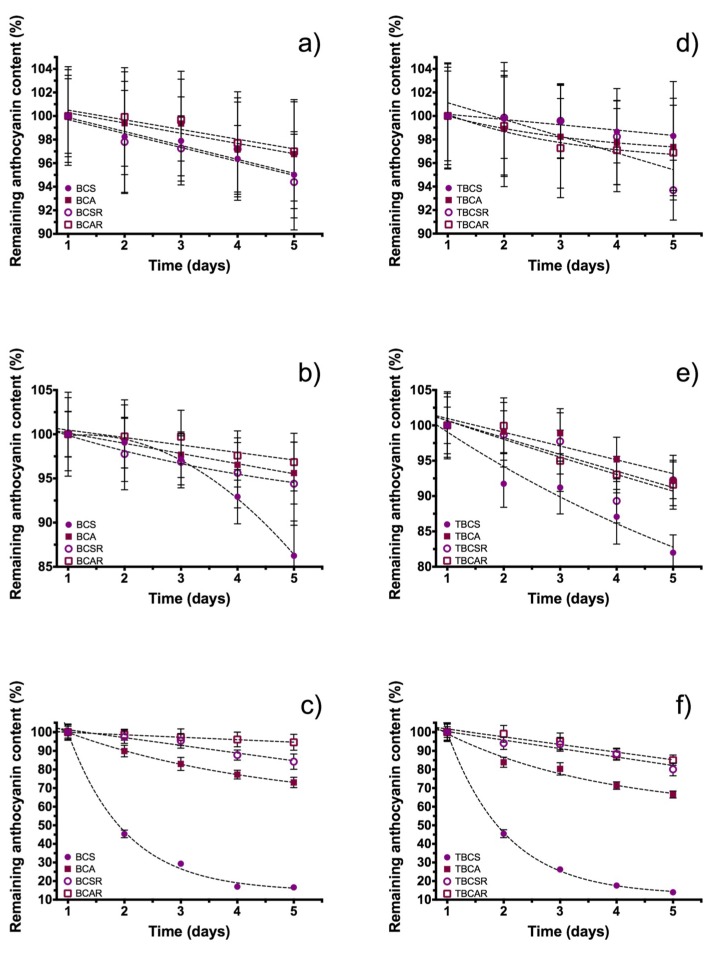
Anthocyanin content as function of storage time at different pH buffers; (**a**,**d**) pH = 4, (**b**,**e**) pH = 7 and (**c**,**f**) pH = 10. Samples extracted with ethanol/citric acid (BCS) and ethanol/acetic acid (BCA), samples after resin column (BCSR and BCAR), and microencapsulated samples using TEOS (TBCS, TBCA, TBCSR, TBCAR).

The pH changes were evaluated using a short-term storage test during five days for analyzing monomeric anthocyanin content changes at acidic, neutral and alkaline pH and Figure 6 shows these changes.

At acid pH (pH = 4, Figure 6a) there was an anthocyanin content loss of 4.98% and 3.22% for BCS and BCA, respectively, and for resin purified extracts (BCSR and BCAR) the losses were 5.58% and 3.01%. At neutral pH (pH = 7, Figure 6b), the content loss was BCS 13.76%, BCA 4.39%, BCSR 5.60% and BCAR 3.17%, and the major changes for the extracts were at alkaline pH (pH = 10, Figure 6c), since the samples with a higher loss from its initial value were 83.38% and 26.98% for BCS and BCA; at this pH, purified extracts had less degradation with a content loss of 15.57% for BCSR and 5.38% BCAR. 

For microencapsulated samples at acid pH (Figure 6d) there was an anthocyanin content loss of 1.69% and 2.63% for TBCS and TBCA respectively and for TBCSR and TBCAR the loss was 5.36% and 3.10%. At neutral pH (Figure 6e) the content loss was 18.02% for TBCS, 7.8% for TBCAR, 7.7% for TBCSR and 8.39% for TBCAR and finally at alkaline pH (Figure 6f) major changes in content were found, such as 86.07% for TBCS, 33.48% for TBCA, 19.90% for TBCSR and 15.02% for TBCAR. Therefore, degradation of microencapsulated samples was reduced at acid pH. When the pH increases, degradation increases too due to the increase in alkalinity. 

In both cases, microencapsulated and non-microencapsulated samples, the graphical tendency is the same and anthocyanins in BCAR samples were the most stable after the elapsed time under three-different conditions of pH, indicating that the anthocyanins extracted with ethanol/acetic acid are more stable to pH changes in comparison with the ethanol/citric acid extracts (higher anthocyanin content loss), which is highly evident at pH = 10.

Since the results from degradation studies of samples microencapsulated with TEOS showed no significant improvement in the stability of the extracts (except for the experiment at 60 °C), these samples were not analyzed for antioxidant activity and color in the food models. This decision was made considering also that the activity of nutraceutical compounds was reduced after treatment [3].

### 2.5. DPPH and Electrochemical CUPRAC Antioxidant Content Test

**Figure 7:**
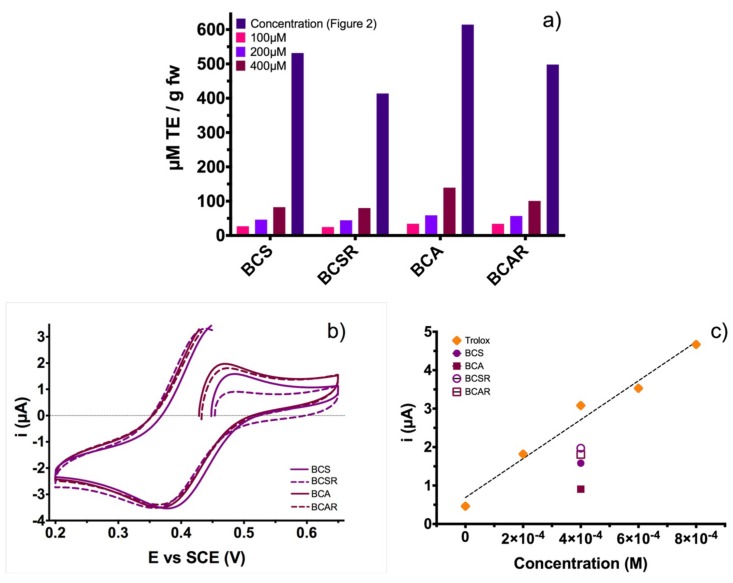
(**a**) Antiradical activity of black carrot extracts using DPPH assay at different concentrations. (**b**) Cyclic voltammograms for the four extracts (400 µM) in a CUPRAC solution. The scan began at the open circuit potential (OCP) with a sweep velocity of 100 mV·s^−1^. (**c**) Electrochemical antioxidant activity and compared with Trolox activity at the same concentration. Samples extracted with ethanol/citric acid (BCS) and ethanol/acetic acid (BCA), samples after resin column (BCSR and BCAR).

As seen from Figure 7a there is a direct relationship of anthocyanin content and antiradical activity; when the anthocyanin content is higher, the antioxidant effect increases. With more phenolic compounds, such as anthocyanins, a higher antiradical activity is expected. 

The extract obtained with acetic acid leads to a higher yield of antiradical activity (614.52 µM TE g fw^−1^) which is 15.5% higher compared with the extract obtained with citric acid. This was expected because the acylated nature of the extracted anthocyanin [25] confers higher antiradical activity than monomeric anthocyanins; also, the use of XAD7 resin reduced antiradical activity on 20.20% and 18.94% for BCS and BCA extracts, respectively.

In the case of CUPRAC test, Figure 7b shows cyclic voltammograms for the antioxidant agents obtained with different extraction methods. It can be observed that the initial potential for the BCAR and BCA was 0.433 and 0.428 V, respectively. Those values exhibited a shift to negative potentials compared with 0.454 and 0.488 V of BCSR and BCS, respectively. This shift to potential negative values is related with an increment in the amount of the complex $Cu{\left(Nc\right)}_{2}^{+}$ due to the capability of the antioxidant agent to donate an electron to the oxidized complex $Cu{\left(Nc\right)}_{2}^{2+}$ according to the following equation:

(1) $$\;aCu{\left(Nc\right)}_{2}^{2+}+bAO_{red}\;↔cCu{\left(Nc\right)}_{2}^{+}+dAO_{ox}^{+}\;$$

where the $AO_{red}$ is the reduced antioxidant agent and the $AO_{oxi}^{+}\;$ is the antioxidant agent when it was oxidized. This behavior is directly related with the antioxidant agent capability of the samples to promote the reduction reaction for the molecule that was previously oxidized $\left(Cu{\left(Nc\right)}_{2}^{2+}\right)$*.* Also, the description above was based on the Nernst equation:

(2) $$\;E=\;E^{0}+\frac{RT}{F}\ln\frac{a_{Cu{\left(Nc\right)}_{2}^{2+}}}{a_{Cu{\left(Nc\right)}_{2}^{+}}}\;$$

where the potential of the reaction on the equilibrium were shifted to negative values owing an increase in the activity (concentration of $Cu{\left(Nc\right)}_{2}^{2+}$) of the products.

Furthermore, the peak current for the oxidation process in the voltammograms has a direct relation with the concentration of $Cu{\left(Nc\right)}_{2}^{+}$ because if the electrolyte has a higher amount of $Cu{\left(Nc\right)}_{2}^{+}$ we obtained a major amount of oxidizer molecules ($Cu{\left(Nc\right)}_{2}^{2+}$) resulting in an increment of the current value. This is possible because the current is directly proportional for the concentration of the species in the reaction (i∝C) [26,27].

The Trolox calibration curve was obtained from the oxidation peak current and was used to analyze the antioxidant activity for the black carrot extracts obtained by the different extraction methods (*r*^2^ = 0.9768). It is proposed that ratio between current peak of the black carrot and the current peak of Trolox (at same concentration, 400 µM) allows determining the antioxidant capability of each black carrot extract. This method is similar to calculating the Trolox Equivalent Antioxidant Capacity (TEAC), where calculations were made by the ratio of molar absorptivity of problem species and Trolox under the corresponding conditions [28]. In order to have cyanidin 3-O-glucoside at a 400 µM concentration, samples were diluted (Figure 3a) and the molecular weight of the anthocyanin was used for the analytical pH differential method.

The calculated Electrochemical Antioxidant Capacity (EAC) is shown in Figure 7c, giving the following results: 0.684, 0.346, 0.748 and 0.591 for BCS, BCA, BCSR and BCAR, respectively. These EAC results exhibited a behavior in accordance with the DPPH colorimetric method. BCA extract had the highest antiradical activity of all samples, followed by BCS, then BCAR and finally BCSR. Also, analyzing the antioxidant activity for black carrot extracts, values of current were under the values of the Trolox calibration plot. The decrease of the antiradical activity is not as higher than the anthocyanin content in Figure 3a; this is because electrochemical CUPRAC methods measure compounds related with the antiradical activity at an electron level. These CUPRAC test shows that our samples could have several 3,7-diglucoside derivatives and other phenolic compounds. The antiradical activity of black carrots extracts was higher than other reported values of several extracts [29].

### 2.6. Color of Black Carrot Extracts on Food Models

**Figure 8:**
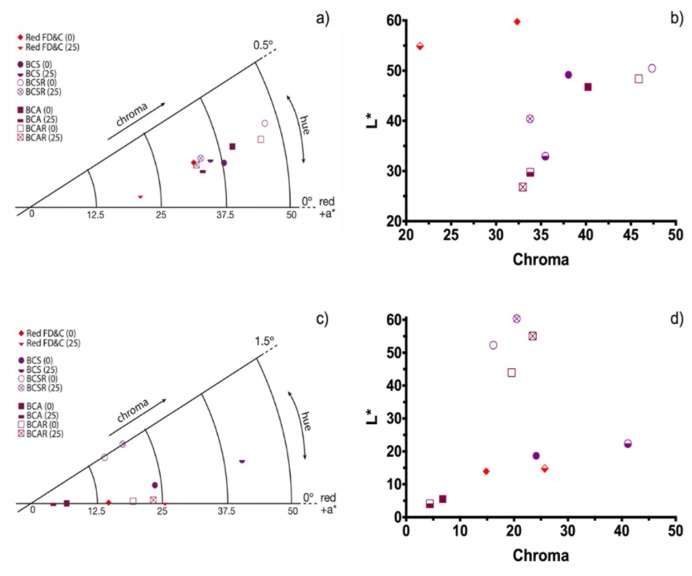
(**a**,**c**) Sectional polar and (**b**,**d**) cartesian diagram of color for the black carrots extracts and the red FD&C on food models (yogurt and jelly) at 0 and 25 days of storage.

Figure 8a−d show the average results from the image analysis for food models using the black carrot extracts and Red FD&C analysis for comparison. All samples (in yogurt and jelly), had a light brown color tendency (hue angle below 2°) at the beginning of the experiment. The jelly has more saturated colors than yogurt and this is due to the base color of the food models (white vs. pale yellow). Red FD&C had the highest luminosity in yogurt and is more saturated than BCS and BCA samples (chroma value). FD&C in jelly is darker, but has the same color saturation than BCS, i.e. in the food model; FD&C and BCS have the same color saturation for the human eye. BCA sample is less saturated in jelly. In the case of BCSR and BCAR, in yogurt, the color is less saturated but darker; and for jelly, they have almost the same saturation (slightly less saturated) but it has a much brighter color.

As seen in Table 2, after the 25-day storage time the color differences (ΔE) in yogurt, of all samples, have values higher than five, which indicates that the color difference at the beginning and after the elapsed time is visually evident; also, samples have higher saturation (lowest chroma value) after storage and specifically the black carrots extract samples get darker since L* value is lower. BCS samples showed similar color difference after storage time compared with red FD&C.

For jelly samples, only sample BCA has a value lower than five, which indicates that the color difference could be distinguished but was not as evident as the rest of the samples; also, BCA samples are the only ones with different saturation and luminosity trends, they had lower chroma values and are darker. The rest of the samples are less saturated (higher chroma value) and brighter. The samples with resin purification (BCSR and BCAR) had a similar color difference compared to red FD&C, except BCA.

For yogurt, BCSR and BCAR samples have the highest color change indicating that cyanidin-3-glucoside derivative anthocyanins are not suitable to be used in this food model, because the acetate group caused important appearance differences under these conditions; for jelly, BCS and BCAR had the highest color changes but BCSR and BCA the lowest compared with Red FD&C. Pigment concentration of the samples must change for the specific commercial use, for instance, strawberry yogurt samples have different L*, a* and b* values [3].

## 3. Materials and Methods 

Fresh black carrot (*Daucus Carota* var. *L.* ssp. *sativus* var. *atrorubens* Alef.) were cultivated in Tlaxcala, Mexico and donated by a local cultivator, then were washed with tap water and stored in 3 kg perforated plastics bags and kept at −20 °C until further use. All reactants used were analytical grade. Ethanol 99.5%, citric acid 99.5%, Amberlite XAD7 resin, tetraethyl orthosilicate (TEOS) 98%, potassium chloride, 6-hydroxy-2,5,7,8-tetramethylchromane-2-carboxylic acid (Trolox), and 2,2-diphenyl-1-picrylhydrazyl (DPPH), CuCl_2_·2H_2_O (99%), neocuproine, and ammonium acetate (98.0%) were supplied by Sigma Aldrich (Toluca, Mexico). Acetic acid, sodium acetate, hydrochloric acid, buffer solutions (pH: 4, 7 & 10) and potassium hydroxide were supplied by J.T. Baker (Mexico City, Mexico). Methanol was supplied by Macron (Hamilton, PA, USA). All experiments were carried out using distilled water obtained from an Elix Advantage Water Purification System (Queretaro, Mexico).

### 3.1. Anthocyanins Extraction and TEOS Microencapsulation

**Figure 9:**
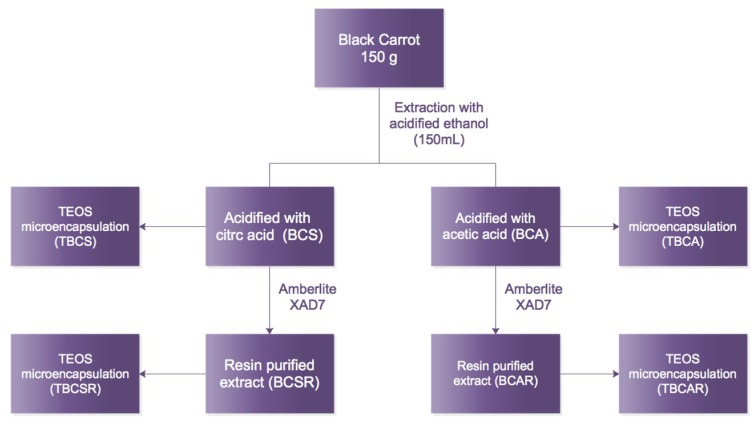
Flow diagram of experimental processes for obtaining samples. Samples extracted with ethanol/citric acid (BCS) and ethanol/acetic acid (BCA), samples after resin column (BCSR and BCAR), and microencapsulated samples using TEOS (TBCS, TBCA, TBCSR, TBCAR).

Acidified ethanol was used as extraction solvent for all samples. Anthocyanins were obtained by blending 150 mL of acidified ethanol (citric/acetic acid solution 85:15 *v*/*v*) and 150 g of sliced frozen carrots (without thawing) with a Grinder 6807 blender Oster (Mexico) for 20 min. Solids were removed by filtration using a 100-mesh sieve filter. The liquid phase (extract) was labeled as BCS (acidified with citric acid) and BCA (acidified with acetic acid). Extracts BCS and BCA were introduced, separately, to a resin column containing Amberlite XAD7 resin for removing non-aromatic compounds from the extract. Flow rate was 32 mL/min (20BV/h) and a solution of 95 mL of ethanol and 5 mL of acidic water (pH = 1.4) was used as eluent. Finally, solvent excess was evaporated at 40 °C using vacuum (R-100, Büchi, Mexico). Samples obtained after this procedure were labeled BCSR and BCAR. All samples were stored in sealed amber glass vials at 4 °C until further use. Then samples were submitted to microencapsulation using TEOS with a procedure reported elsewhere [1,3]. The samples after microencapsulation were labeled as TBCS, TBCA, TBCSR and TBCAR. A flow diagram is shown in Figure 9.

### 3.2. Anthocyanin Content

It has been reported that black carrot contains different monomeric anthocyanins with different sugar moieties such as peonidin, pelargonidin and cyanidin, being cyanidin 3-O-R the major compound in the total anthocyanin content [20,21,22,23]. In order to confirm this and to justify the use of cyanidin 3-O-glucoside for analytical measurements, Fourier Transform Infrared Spectroscopy (FTIR) spectra under attenuated total reflectance (ATR) was measured by a Frontier MIR/NIR Spectrometer (Perkin Elmer, Waltham, MA, United States), for dried samples of BCA and BCS and then compared with a frequency quantum chemical calculation of the same molecule (Figure 1c).

For the frequency calculation of cyanidin 3-O-glucoside molecule a personal computer running Gaussian 98W [30] was used. The geometry was fully optimized assuming Cs point group symmetry using the Becke 3-Lee-Yang-Parr (B3LYP), supplemented with the standard 6-31 + G basis sets. The simulated IR spectra were plotted using Avogadro molecular viewer [31] and the vibrational modes were analyzed and compared to the experimental data as mentioned.

Total anthocyanins content (monomeric anthocyanins) was determined using the pH-differential method reported by Giusti and Wrolstad [32], using a molar extinction coefficient of 26900 M^-1^cm^-1^ that corresponds to cyanidin 3-O-glucoside. The average molecular weight used was 756.87 g mol^-1^ of the anthocyanins according to previous studies from black carrot [32,33,34]. A VWR 1600-PC spectrophotometer and 1 cm path length glass cells were used; measurements were performed scanning from 700 to 400 nm at room temperature (~24 °C). Finally, High pressure liquid chromatography (HPLC) was performed on a Flexar LC (Perkin Elmer) system using a 250 mm × 4.6 mm C18 reverse phase column and BCS and BCA samples were measured according to conditions reported elsewhere [34,35]; using a flow rate of 1.0 mL/min and the chromatographs were recorded at 520 nm using PDA Plus Detector coupled with the equipment.

### 3.3. Degradation Studies

Black carrot extracts were transferred into vials with screw caps to perform degradation tests. All experiments were performed in triplicate and the referred anthocyanin content was normalized to 200 mg/L in all the samples in order to make direct comparison between samples so the results are presented as remaining percentage of cyanidin 3-O-glycoside. 

The ASTM D 4320 method was used to determine anthocyanin stability against UV radiation. Black carrot extracts were exposed to UV lamp irradiation (315–400 nm) for 160 min; samples were placed within 15 cm from the source. Measurements were taken every 40 minand the temperature was kept constant at 25 °C under a working area of 3.5 m^2^ isolated from other light sources [1,3]. 

For thermal stability studies, samples were placed in a preheated water bath at 80 °C, 60 °C and 40 °C. Samples were removed from water bath every 20 min, up to 120 minand rapidly cooled to room temperature. Anthocyanin content was analyzed immediately [35]. 

The anthocyanin stability in storage was also studied at three different pH conditions (4, 7 and 10) at room temperature (~24 °C). For this purpose, 3 mL of phosphate buffer solution were prepared at the required pH conditions and then colored with 500 µL of black carrot extract concentrate; these colored solutions were used without further treatment. Finally, anthocyanin storage stability was determined every 24 h during 5 days for each of the black carrot extracts [12].

### 3.4. Antioxidant Activity 

The DPPH radical scavenging activity assay was performed according to several methods described previously [22,36,37,38] in which radical scavenging activities were determined by testing the extracts with the free radical DPPH and monitoring their absorbance decrease at 515 nm using a 1600-PC spectrophotometer (VWR, Graumanngasse, Vienna) with a 1 cm path length glass cells. Control assays using the black carrot extracts were performed in order to obtain their absorbance contributions. A solution of 50 µM DPPH was prepared using buffered methanol, which was prepared by mixing methanol with acetic acid buffer solution (0.1 M, pH 5.5) [36]. Then, 2.85 mL of DPPH solution were mixed with 150 µL of each extract and were left reacting 30 minat room temperature (~24 °C). Antiradical activity was expressed as Trolox equivalents per gram of fresh weight (µM TE g·fw^−1^), which was calculated from the equation obtained using a linear regression after plotting the known absorbance with different Trolox concentrations, from 1 to 800 µM and *r*^2^ = 0.9657. 

A cupric reducing antioxidant capacity (CUPRAC) solution [24,26] was prepared to determine antioxidant capacity via electrochemical tests using CuCl_2_ with a concentration of 3 mM in distilled water. Also, a solution of neocuproin at 6 mM in ethanol was prepared. In order to control pH of the main solution, a 1.2 M ammonium acetate buffer solution was prepared (pH = 7), then pH was adjusted adding 1.2 M HCl and 1.2 M NaOH as required. Concentrations of Trolox were varied from 1 to 800 µM in ethanol for obtaining a Trolox standard curve. 2 mL of each CuCl_2_, neocuproin, ammonium acetate buffer, Trolox and distilled water solutions were prepared and mixed. The 10 mL solution was stirred for 15 minand N_2_ was bubbled into it for 5 min. The same procedure was followed to evaluate antioxidant capacity adding anthocyanins extracts instead of Trolox solution and the values are reported as a comparison between the analytical response vs. concentration plot during the antioxidant quantification because the slope is dependent on the stochiometric relationship between the antioxidant-oxidant species involved, which is related to the electron transfer per molecule pair.

The electrochemical tests were performed in a three-electrode electrochemical cell. A calomel Hg/Hg_2_Cl_2_ (saturated with KCl) was used as reference electrode, a graphite bar was used as counter electrode and a glassy carbon (3 mm) electrode was used as working electrode. Before each electrochemical measurement, the working electrode was polished with aluminum oxide powder followed by ultrasonic stirring during 10 min; this process was repeated 3 times. The voltammograms were obtained in a Bio-LogicVP-50 potentiostat (Bio-Logic Science Instruments, Seyssinet-Pariset, France) with a sweep velocity of 100 mV s^−1^, starting the voltammograms from the open circuit potential (OCP) that was determined when the potential did not show a variation higher than 1 mV per second.

### 3.5. Extract and Food Models Color Determination

A custom MATLAB script (Mathworks Inc., Natick, MA, USA) was used to measure lightness and chromaticity coordinates in the L* a* b* color space (CIELAB) according to CIE standard illuminant A (typical, domestic, tungsten-filament lighting with correlated color temperature of 2856 K). L* indicates lightness, a* and b* are chromaticity coordinates, h (hue), c (chroma) and ΔE (color change) were calculated from a* and b* values. Digital images from samples were taken using a Sony digital camera α99II coupled with a Vario Sonnar T* 24–70 mm lens (Sony Corporation, Tokyo, Japan) under the same light conditions; the images were cropped to 1024 × 1024 pixels and then processed with the afore mentioned script. Additionally, samples were measured in two different colored food models: yogurt and jelly. For yogurt food model, 10 g of commercial yogurt (Yoplait natural yogurt, Sigma Alimentos Lácteos México, Queretaro, Mexico) were colored using 10 mg of calculated anthocyanins from each extract and jelly was prepared using jelly powder (Coloidales Duche, Ciudad de México, Mexico) dissolved in boiling water (1:3 ratio) and 10 g of the mixture were colored using the same calculated amount of anthocyanins from each extract. Samples were measured at the beginning of the experiment and after 25 days, and compared with a colored yogurt/jelly with 10 mg of Red FD&C (Red Currant 12.5%, Colores Duche, Ciudad de México, Mexico) under the same conditions. 

## 4. Conclusions

Anthocyanins were extracted from black carrot with ethanol/citric acid and ethanol/acetic acid for comparison between total anthocyanin content, and stability against media changes and antioxidant capacity was obtained to analyze samples in food models. Microencapsulation with TEOS was performed with the objective of enhancing anthocyanin stability. Extracts had the highest degradation in alkaline pH, and BCAR was the most stable sample to pH media changes. The antiradical activity of black carrots extracts was higher than other reported values, and when anthocyanin content is higher, the antioxidant effect increases. Results of UV radiation and thermal stability tests indicate that TEOS microencapsulation provides a negligible improvement in anthocyanins’ stability. In conclusion, extraction with ethanol/acetic acid is the most convenient and stable treatment against pH media changes. Purification with resin and TEOS microencapsulation did not increase stability of the black carrot extracts. TEOS microencapsulation has proven to be effective (enhancing stability) for some dried materials from natural extracts in our previous research, but we do not recommend its use for materials obtained from black carrot extracts. Even though anthocyanins are already used in the food industry in beverages, our samples were not suitable for the yogurt or jelly model selected except for BCA sample in jelly that has the highest antioxidant activity, this gives it potential for being a functional natural colorant in this specific food model. 

## Figures and Tables

**Table 1:** Retention time and anthocyanin identification from black carrot extracts BCS (extracted with ethanol/citric acid) and BCA (extracted with ethanol/acetic acid).

Peak	Retention Time (min)		Anthocyanin
	BCS	BCA	
1	10.94	10.93	Cyanidin-3-xylosyl-glucosyl-galactoside
2	14.46	14.39	Cyanidin-3-xylosyl-galactoside
3	16.91	16.88	Sinapic acid derivative of cyanidin 3-xylosyl-glucosyl-galactoside
4	19.43	19.42	Ferulic acid derivative of cyanidin 3-xylosyl-glucosyl-galactoside
5	20.45	20.44	Coumaric acid derivative of cyanidin 3-xylosyl-glucosyl-galactoside
6	-	23.98	Feruic acid derivative of pelargonidin 3-xylosyl-glucosyl-galactoside
7	-	25.17	Ferulic acid derivative of peonidin 3-xylosyl-glucosyl-galactoside

**Table 2:** Color comparison between food models (yogurt and jelly) using black carrot extracts after 25 days in storage.

Sample	ΔE Yogurt	ΔE Jelly
Red FD&C	12.62	10.92
BCS	16.45	17.61
BCSR	24.27	9.21
BCA	18.31	2.82
BCAR	25.12	12.71
